# Indole-3-Acetic Acid Production by *Klebsiella variicola* from Watermeal and Its Role in Enhancing Watermeal Growth

**DOI:** 10.3390/plants15172642

**Published:** 2026-08-28

**Authors:** Tanaporn Ladthaisong, Thanawan Gateta, Wasan Seemakram, Urachart Kokaew, Nisachon Jangpromma, Sophon Boonlue

**Affiliations:** 1 Department of Microbiology, Faculty of Science, Khon Kaen University, Khon Kaen 40002, Thailand; tanaporn.l@kkumail.com (T.L.); thanawan.gateta@kkumail.com (T.G.); 2 Department of Microbiology and Parasitology, Faculty of Medical Science, Naresuan University, Phitsanulok 65000, Thailand; wasans@nu.ac.th; 3 Applied Intelligence and Data Analytics Laboratory, College of Computing, Khon Kaen University, Khon Kaen 40002, Thailand; urachart@kku.ac.th; 4 Protein and Bio-Innovation Research Center for Sustainable Health (ProBISH), Khon Kaen University, Khon Kaen 40000, Thailand; nisaja@kku.ac.th; 5 Department of Biochemistry, Faculty of Science, Khon Kaen University, Khon Kaen 40000, Thailand

**Keywords:** endophytic bacteria, indole-3-acetic acid, plant protein, response surface methodology, *Wolffia* spp.

## Abstract

Plant growth-promoting bacteria (PGPB) enhance plant development through mechanisms such as the production of indole-3-acetic acid (IAA). However, endophytic bacteria associated with aquatic plants, particularly watermeal (*Wolffia* spp.), remain poorly explored. This study investigated the potential of bacterially derived crude IAA extract for enhancing watermeal growth and accumulation of secondary metabolites in watermeal. This study isolated and screened endophytic bacteria from watermeal for IAA production, identifying *Klebsiella variicola* W1/6 as the most efficient producer. Optimization using response surface methodology (RSM) revealed optimal conditions at pH 5.84, 5 days of incubation, and 10.63% (*v*/*v*) inoculum size. Crude IAA extract significantly enhanced watermeal growth under both laboratory and greenhouse conditions. Notably, the combination of 0.5 ppm crude IAA extract with 50% chemical fertilizer produced growth comparable to that of the full fertilizer treatment, indicating reduced fertilizer requirement. These findings highlight the potential of bacterially derived IAA from *K. variicola* W1/6 as a microbial biostimulant for sustainable aquatic plant cultivation.

## 1. Introduction

Enhancing the production efficiency of small aquatic plants such as watermeal (*Wolffia* spp.), commonly known as “pham” in Thai, has gained increasing attention in recent years. Watermeal is a high-nutritional-value aquatic plant, especially rich in protein, which can comprise up to 40% of its dry weight [1]. Watermeal is a good source of important amino acids, vitamins, and minerals for both people and animals [2]. It grows fast and can be grown in shallow water without needing a lot of land. This makes it a promising alternative food source. Watermeal can also be used to treat wastewater, produce biomass, and serve as a raw material for industries. Despite its high potential, large-scale cultivation of Watermeal still faces challenges, especially in controlling its growth rate. Growth depends on factors such as light intensity, temperature, nutrient levels in water, and plant hormones that regulate cell division and expansion, which directly affect biomass production [3]. Indole-3-acetic acid (IAA) is an important auxin-type phytohormone that significantly influences plant growth and development, particularly in processes like root initiation, cell elongation, and cell division [3]. Although plants can naturally synthesize IAA, microbial production of IAA has attracted increasing attention as a biological and potentially more sustainable approach for agricultural applications. Various microorganisms, especially plant growth-promoting bacteria (PGPB), are capable of producing IAA and have been widely studied for their ability to enhance plant growth [4]. PGPB produce IAA through tryptophan-dependent pathways. However, their application in aquatic plants remains poorly explored. Bacterially derived IAA has attracted increasing attention as a sustainable alternative to synthetic auxins because it can be produced through microbial fermentation at lower cost and with reduced environmental impact. Prior research has shown that microbial IAA can stimulate root formation, enhance agricultural productivity, and boost plant resilience under stress conditions. Moreover, whether bacterially derived IAA can regulate biomass production independently of root development remains unknown. Since watermeal has minimal or no root structures, it provides a compelling model for studying whether IAA contributes to biomass growth or stimulates cell division.

Although *Wolffia* spp. can synthesize endogenous indole-3-acetic acid (IAA), plant-associated endophytic bacteria may provide an additional source of phytohormones that can enhance plant growth and improve nutrient utilization. Therefore, the isolation of IAA-producing endophytic bacteria from *Wolffia* may offer an alternative strategy for promoting plant growth while reducing dependence on chemical fertilizers. The objectives of this study were to isolate and identify efficient IAA-producing endophytic bacteria associated with *Wolffia*, optimize bacterial IAA production, and evaluate the potential of bacterially derived crude IAA extract to enhance growth, physiological performance, and secondary metabolite accumulation in *Wolffia* spp. under controlled cultivation conditions. In addition, the ability of the crude IAA extract to reduce chemical fertilizer requirements and function as a microbial biostimulant for sustainable watermeal cultivation was assessed.

## 2. Results

### 2.1. Isolation, Screening, and Identification of Bacteria

#### 2.1.1. Isolation of Bacteria

A total of 35 bacterial isolates were obtained from watermeal (*Wolffia* spp.). Watermeal samples were obtained from farms in Kalasin province, Roi Et province, and Bangkok province, Thailand. All the bacterial isolates were screened for their ability to produce indole-3-acetic acid (IAA).

#### 2.1.2. Screening of IAA Production

A total of 35 bacterial isolates were screened for their ability to produce IAA. Among these, four isolates produced relatively high levels of IAA (≥50 µg/mL). The highest IAA production was observed in isolate W1/6, with an IAA concentration of 106.34 µg/mL, followed by isolates WT1/3, W2/1, and W4/4, which produced 87.47, 62.23, and 58.82 µg/mL, respectively. Therefore, isolate W1/6 was selected for further optimization of IAA production using response surface methodology (RSM) and subsequent IAA quantification. (Detailed results are shown in Supplementary Table S1).

#### 2.1.3. Identification of Bacterial

**Figure 1:**
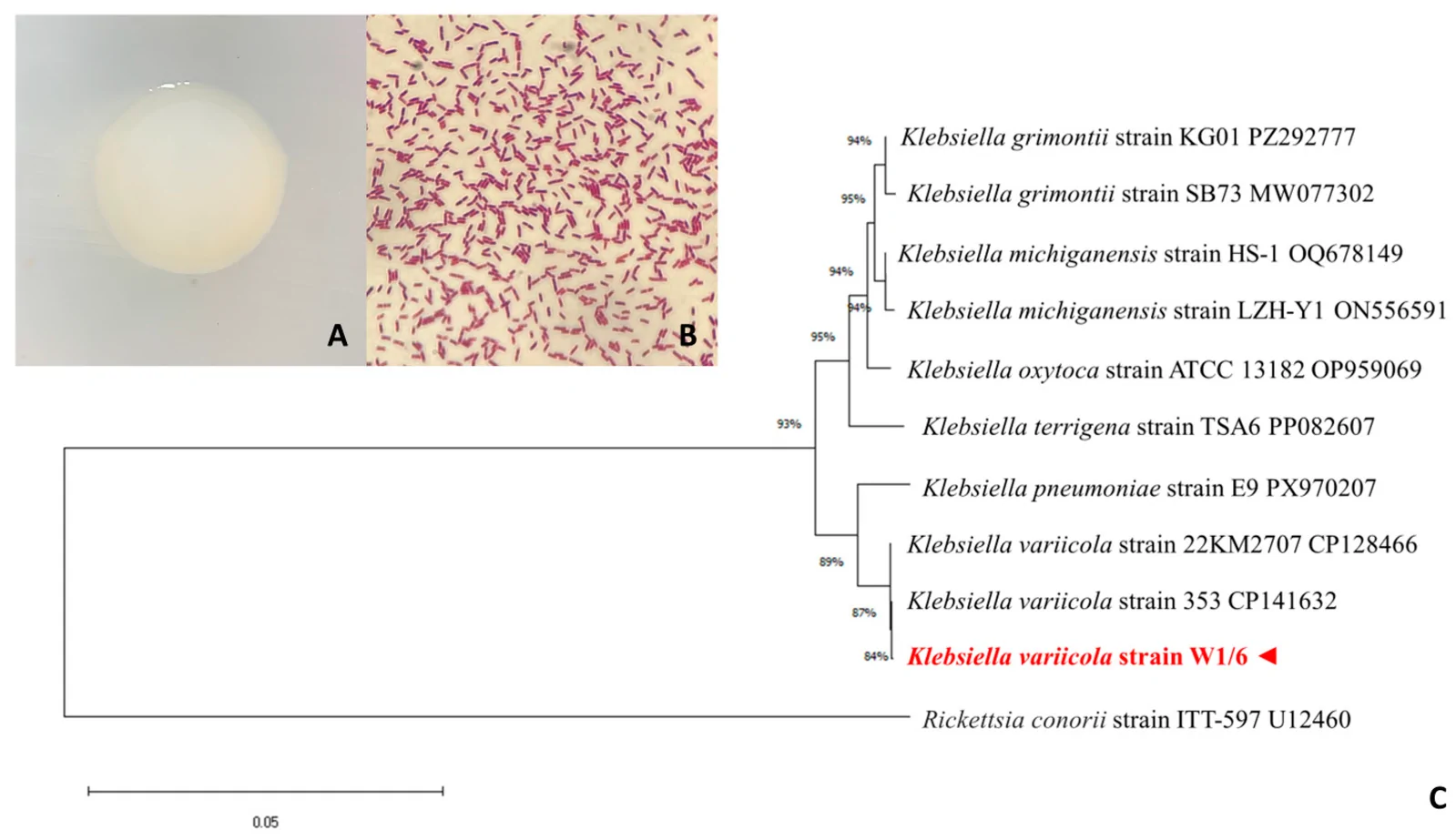
Morphological and molecular characteristics of isolate W1/6: (**A**) colony morphology of isolate W1/6 on nutrient agar; (**B**) Gram-stained cells of isolate W1/6; and (**C**) phylogenetic tree showing the relationship between isolate W1/6 and related *Klebsiella* species. The phylogenetic tree was constructed using the neighbor-joining method with 1000 bootstrap replicates in MEGA 11 software.

The selected isolate W1/6 formed circular, creamy-white, highly mucoid, convex colonies with entire margins on nutrient agar, exhibiting a dome-shaped appearance. Gram staining revealed that the cells were Gram-negative rods occurring predominantly singly or in pairs (Figure 1A,B). Molecular identification based on 16S rRNA gene sequence analysis showed that W1/6 shared 99% sequence similarity with *K. variicola* strains deposited in the NCBI database. Phylogenetic analysis further showed that isolate W1/6 clustered with *K. variicola* reference strains, clearly distinguishing it from other closely related *Klebsiella* species (Figure 1C). The 16S rRNA gene sequence of isolate W1/6 was deposited in GenBank under accession number PZ321724.

### 2.2. Determination of Optimal Conditions for IAA Production Using Response Surface Methodology (RSM)

Response surface methodology (RSM) was applied to determine the optimal levels of initial pH, incubation time, and inoculum size for IAA production using a face-centered central composite design (CCD; α = 1). Analysis of variance (ANOVA) (Table 1) and the experimental design, results (Table 2).

The relationship between the studied factors, including pH (A), incubation time (B), and inoculum size (C), and the response variable, indole-3-acetic acid (IAA) production (Y), was described using a quadratic model. The model illustrates the significant effects of individual factors and their interactions, as expressed in Equation (1).

IAA production (Y) = 156.56 + 12.59A + 17.22B + 12.42C − 17.27AB − 8.73AC − 13.74BC − 4.97A^2^ − 9.31B^2^ − 14.95C^2^ (1)

**Figure 2:**
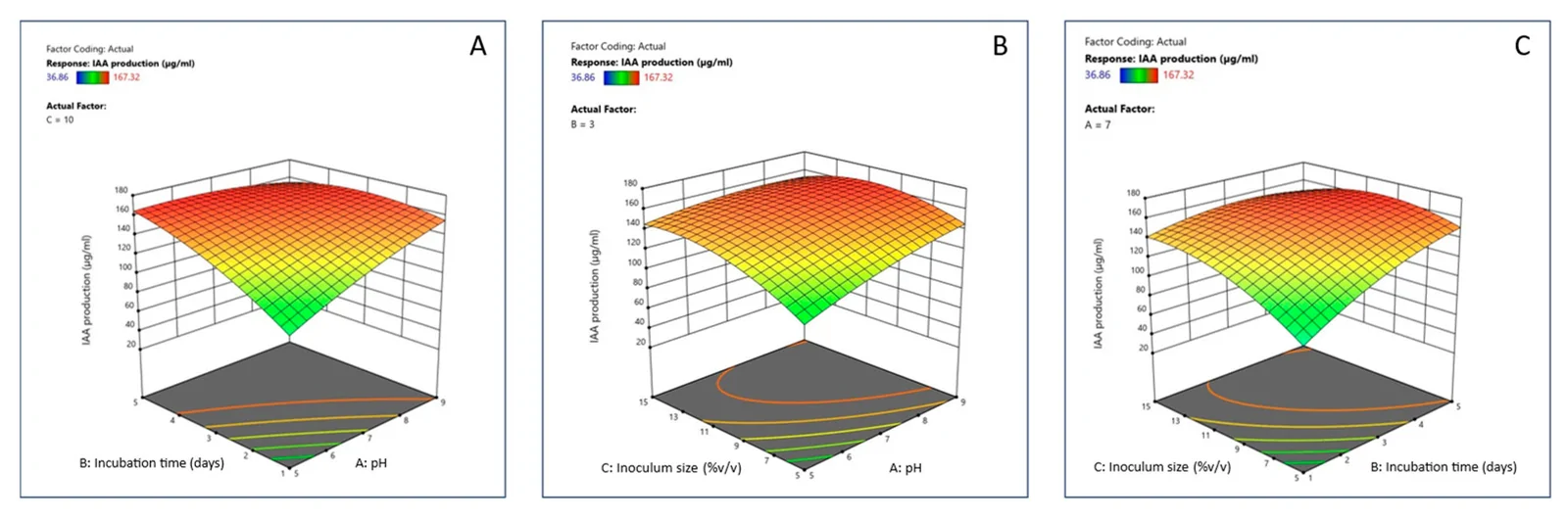
Response surface plots showing the effects of various factors on IAA production by *K. variicola* W1/6: (**A**) effect of incubation time and pH; (**B**) inoculum size and pH; (**C**) inoculum size and incubation time.

Analysis of variance of the quadratic model was statistically significant (F = 9.11 and *p* = 0.0041). The coefficient of determination (R^2^) was 0.9213, indicating that 92.13% of the variation in IAA production was explained by the model. The adjusted R^2^ value was 0.8202, suggesting a satisfactory agreement between the predicted and experimental values. The lack-of-fit test was not significant (*p* = 0.7413), confirming that the model was appropriate and capable of predicting the response with good agreement with the experimental data. Evaluation of the individual model terms revealed that the linear effects of pH (A), incubation time (B), and inoculum size (C) significantly influenced IAA production (*p* < 0.05). In addition, the interaction effects between pH and incubation time (AB) and incubation time and inoculum size (BC) were statistically significant, whereas the interaction between pH and inoculum size (AC) was not significant. The quadratic terms (A^2^, B^2^, and C^2^) did not show significant effects on IAA production. Three-dimensional response surface plots were generated to illustrate the interaction effects of the studied factors on IAA production. IAA production increased when both pH and incubation time were within appropriate ranges, which is consistent with the statistical analysis indicating a significant AB interaction (Figure 2A). An increase in IAA production was observed at suitable inoculum sizes combined with optimal pH values; however, this interaction was not statistically significant (Figure 2B). IAA production markedly increased with longer incubation times in combination with an appropriate inoculum size, in agreement with the statistically significant BC interaction (Figure 2C).

Based on the quadratic model, optimal conditions for maximum IAA production were predicted as pH 5.84, an incubation time of 5 days, and an inoculum size of 10.63% (*v*/*v*), yielding a predicted IAA concentration of 165.73 µg/mL. To validate the model, experiments were conducted under the predicted optimum conditions. The experiment resulted in an IAA concentration of 164.69 ± 0.62 µg/mL, which differed by only 0.63% from the predicted value. This close agreement confirms the accuracy and reliability of the developed RSM model.

### 2.3. Identification of a Bacterial IAA Biosynthesis Pathway

**Figure 3:**
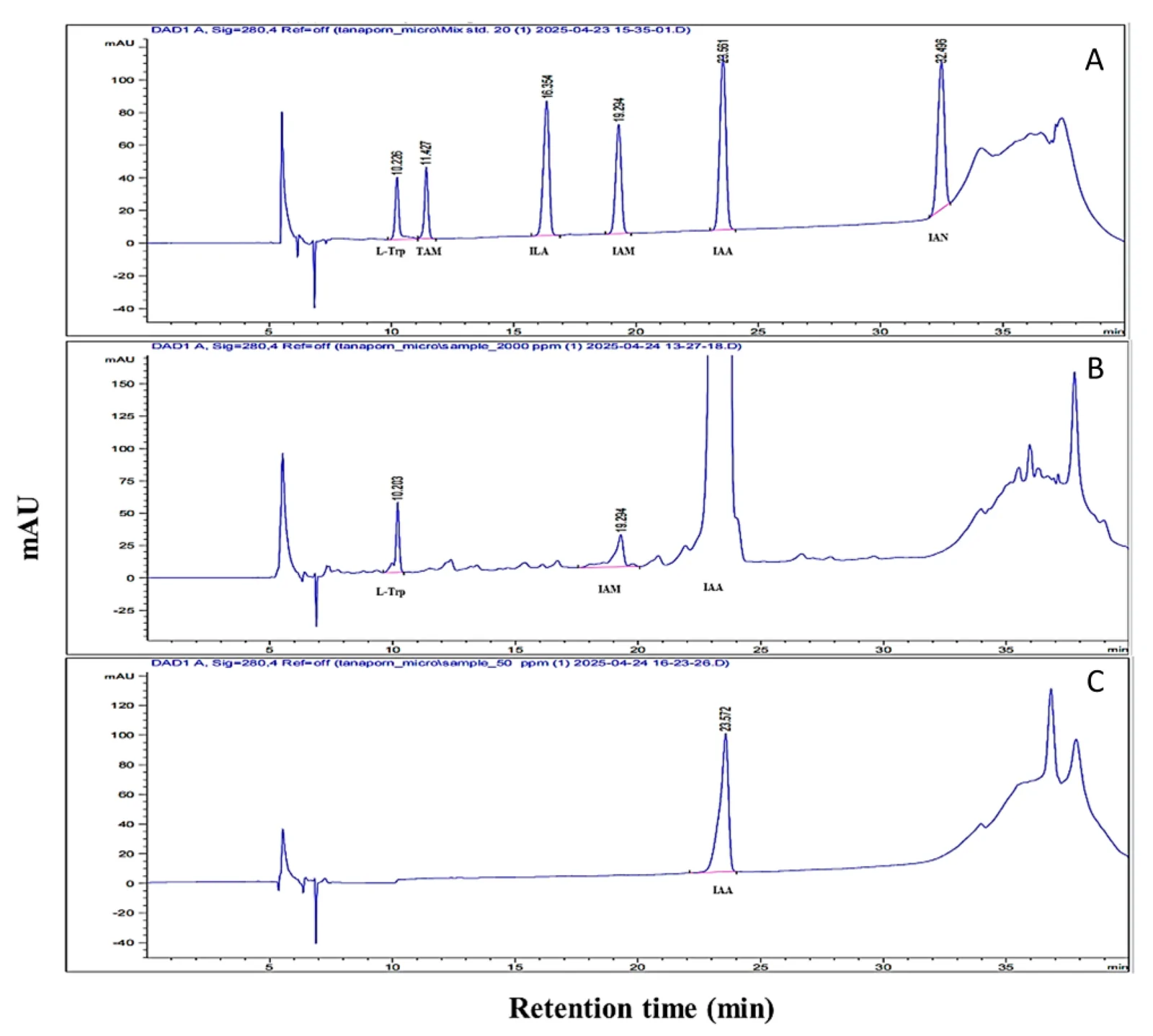
Identification of indole compounds produced by *K. variicola* W1/6 using HPLC analysis: (**A**) mixed indole compounds standard, (**B**) crude extracts of *K. variicola* W1/6 (2000 ppm), (**C**) crude extracts of *K. variicola* W1/6 (50 ppm). L-Trp: L-tryptophan; TAM: Tryptamine; ILA: Indole-3-lactic acid; IAM: Indole-3-acetamide; IAA: Indole-3-acetic acid; IAN: Indole-3-acetonitrile. The y-axis represents detector response expressed as milli-absorbance units (mAU), and the x-axis represents retention time (min).

HPLC analysis of the crude bacterial extract indicated that the isolate was capable of producing IAA via a tryptophan-dependent (Trp-dependent) biosynthesis pathway compared to the standards. In *K. variicola* W1/6, distinct peaks corresponding to IAA, L-tryptophan (L-Trp), and indole-3-acetamide (IAM) were detected, indicating the involvement of the IAM pathway in IAA biosynthesis. In contrast, no peaks corresponding to other indole-related intermediates, including tryptamine (TAM), indole-3-acetonitrile (IAN), or indole-3-lactic acid (ILA), were observed in the crude extracts. These findings suggest that *K. variicola* W1/6 synthesizes IAA predominantly through a Trp-dependent pathway involving IAM as a key intermediate (Figure 3).

### 2.4. The Effect of Combining Chemical Fertilizer with IAA-Containing Crude Extract on the Growth Promotion of Watermeal Under Laboratory Conditions

**Figure 4:**
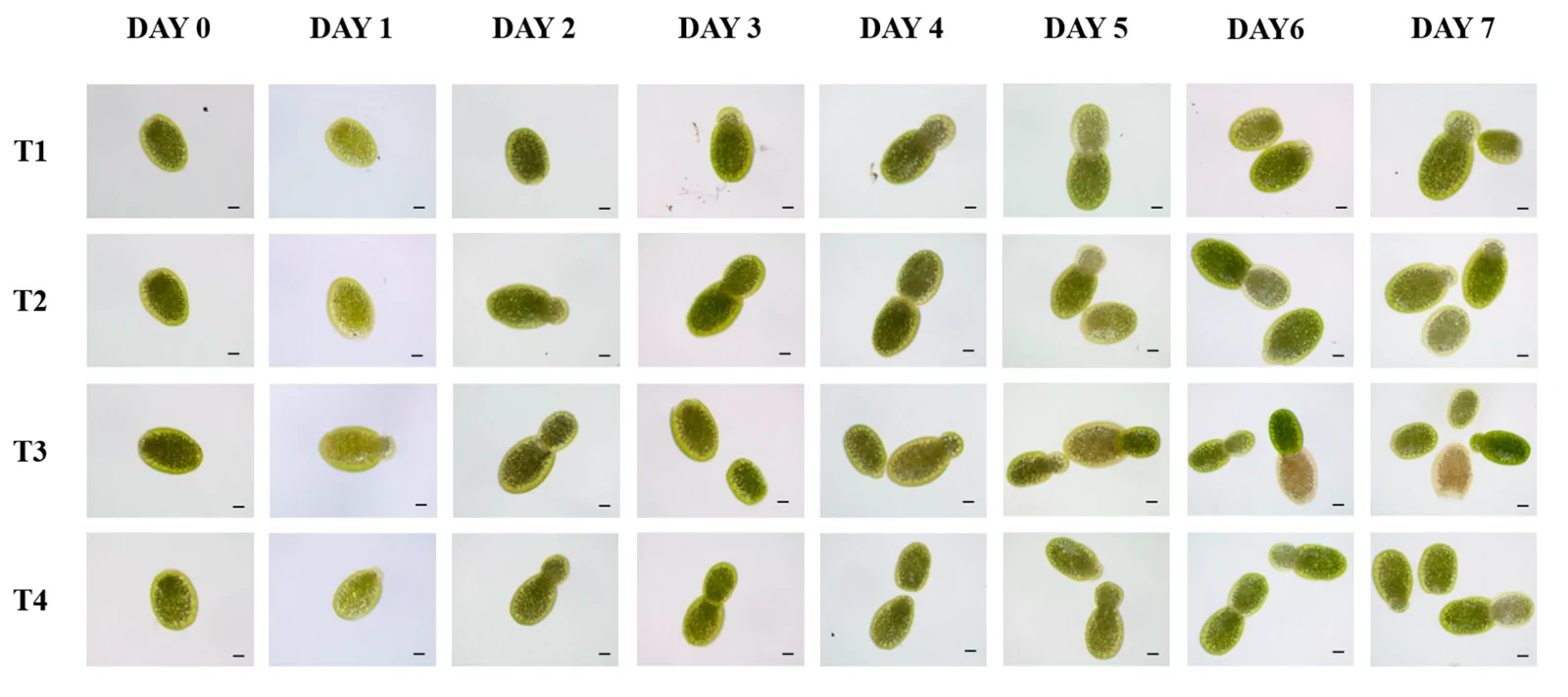
Morphological observations of watermeal under a stereomicroscope. Throughout the cultivation period from day 0 to day 7, scale bars = 0.1 mm. (T1) sterile distilled water (Control), (T2) full dose (100%) of chemical fertilizer (NPK of 15-15-15), (T3) crude extract containing 0.5 ppm IAA and (T4) crude extract containing 0.5 ppm IAA and half dose (50%) of chemical fertilizer.

The crude extract containing IAA was subsequently evaluated for its effects on watermeal growth in combination with different levels of chemical fertilizer. The control treatment produced an average of 47.33 ± 6.81 fronds with an average frond size of 0.58 ± 0.06 mm, whereas application of 100% chemical fertilizer increased the average number of fronds to 74.00 ± 2.65. Treatment with crude extract containing 0.5 ppm IAA resulted in an average frond size of 0.63 ± 0.08 mm. Notably, the combination of 50% chemical fertilizer with crude extract containing 0.5 ppm IAA produced an average of 73.00 ± 4.24 fronds with an average frond size of 0.60 ± 0.05 mm (Table 3). Morphological observations under a stereomicroscope (Figure 4) further supported the quantitative findings. Throughout the cultivation period from day 0 to day 7, treatments supplemented with crude extract, particularly when combined with chemical fertilizer, exhibited a marked increase in daughter frond formation. The combination of 50% chemical fertilizer with crude extract containing 0.5 ppm IAA showed rapid bud emergence and active vegetative propagation. These findings suggest that the combined application of crude extract and 50% chemical fertilizer was able to maintain watermeal growth comparable to the full fertilizer treatment, indicating its potential to reduce fertilizer inputs.

### 2.5. The Effect of Combining Chemical Fertilizer with IAA-Containing Crude Extract on the Growth Promotion of Watermeal Under Greenhouse Conditions

#### 2.5.1. Biomass and Chlorophyll Content

The results demonstrated that chlorophyll content and biomass of watermeal differed significantly among treatments at all the sampling times (Table 4). At 7 days after transplantation (DAT), T2 exhibited the highest chlorophyll a, chlorophyll b, and total chlorophyll contents (21.36 ± 3.17, 13.12 ± 2.89, and 32.63 ± 7.02 mg g^−1^ FW, respectively). In terms of biomass, T4 and T2 produced the highest fresh weight (35.31 ± 1.45 and 33.87 ± 5.01 g tray^−1^, respectively), which were significantly higher than T1 and T3. However, dry weight at this stage was higher in T4, T3, and T1 compared to T2. At 14 DAT, T2 maintained the highest chlorophyll a and total chlorophyll contents (13.78 ± 1.67 and 21.92 ± 1.14 mg g^−1^ FW, respectively). Chlorophyll b in T3 and T4 was comparable to T2 and higher than T1. Biomass followed a similar trend, with T2 and T4 producing the highest fresh weight (68.56 ± 5.38 and 65.24 ± 5.07 g tray^−1^, respectively). These treatments also exhibited the highest dry weight. At 21 DAT, T2 exhibited the highest chlorophyll b and total chlorophyll contents (4.54 ± 1.42 and 10.05 ± 1.07 mg g^−1^ FW, respectively). In contrast, T4 produced the highest fresh and dry weights (78.61 ± 4.75 and 2.53 ± 0.00 g tray^−1^, respectively), which were significantly higher than those of the other treatments. T1 and T3 generally exhibited lower biomass accumulation than T2 and T4.

**Figure 5:**
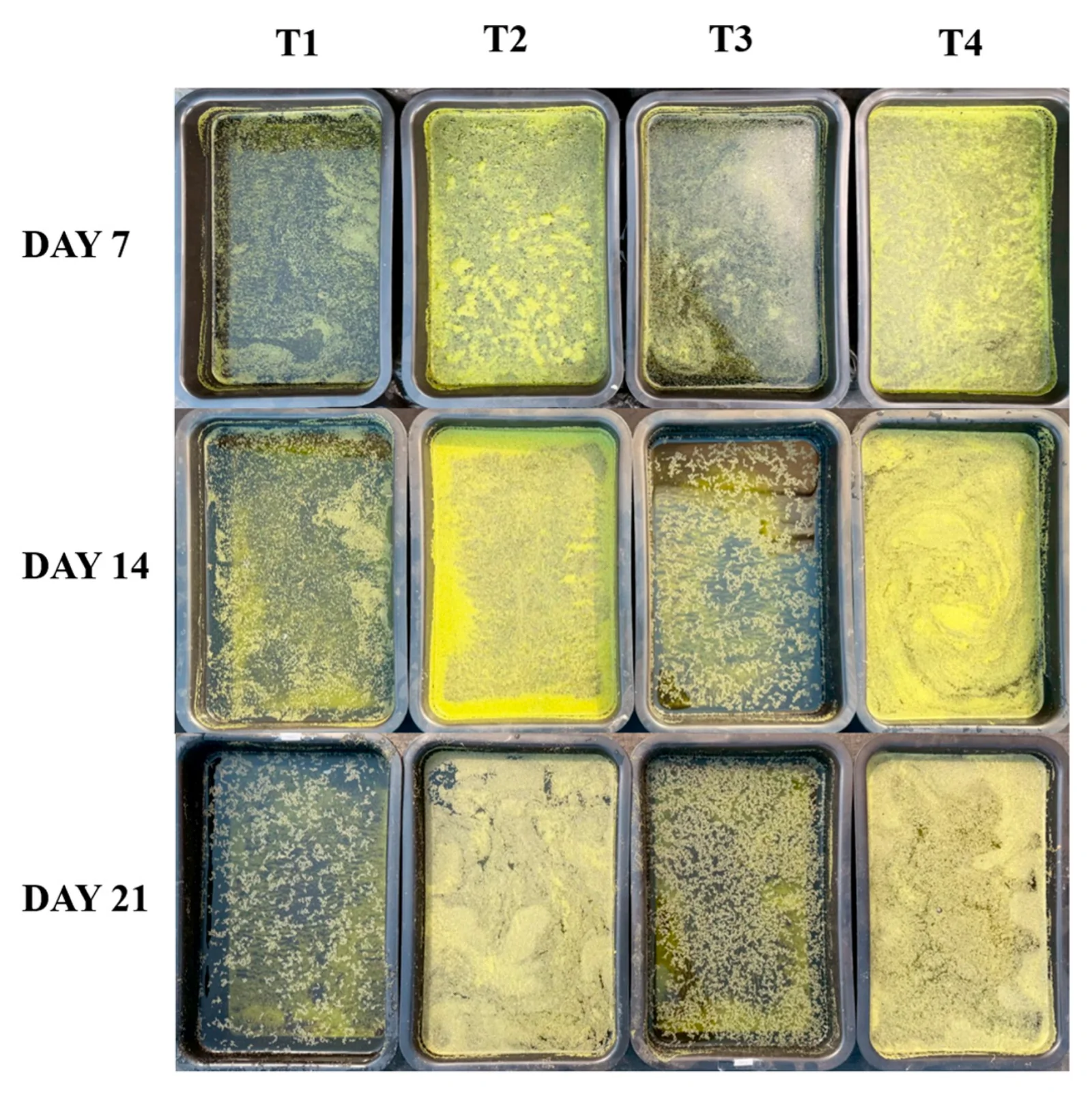
Representative photographs of watermeal growth at 7, 14, and 21 days after treatments (DAT) under greenhouse conditions. (T1) Tap water without fertilizer or crude extract (control), (T2) full dose (100%) of chemical fertilizer (NPK 15-15-15), (T3) crude extract derived from *K. variicola* W1/6 containing 0.5 ppm IAA, and (T4) crude extract derived from *K. variicola* W1/6 containing 0.5 ppm IAA combined with a half dose (50%) of chemical fertilizer. Photographs represent one biological replicate per treatment and sampling time.

The visual observations were consistent with the quantitative data (Figure 5). Treatments T2 and T4 exhibited greater plant coverage and biomass density compared to T1 and T3 throughout the cultivation period. A reduction in green coloration was observed in all the treatments at the later stage, particularly in T1 and T3.

#### 2.5.2. Protein Content

**Figure 6:**
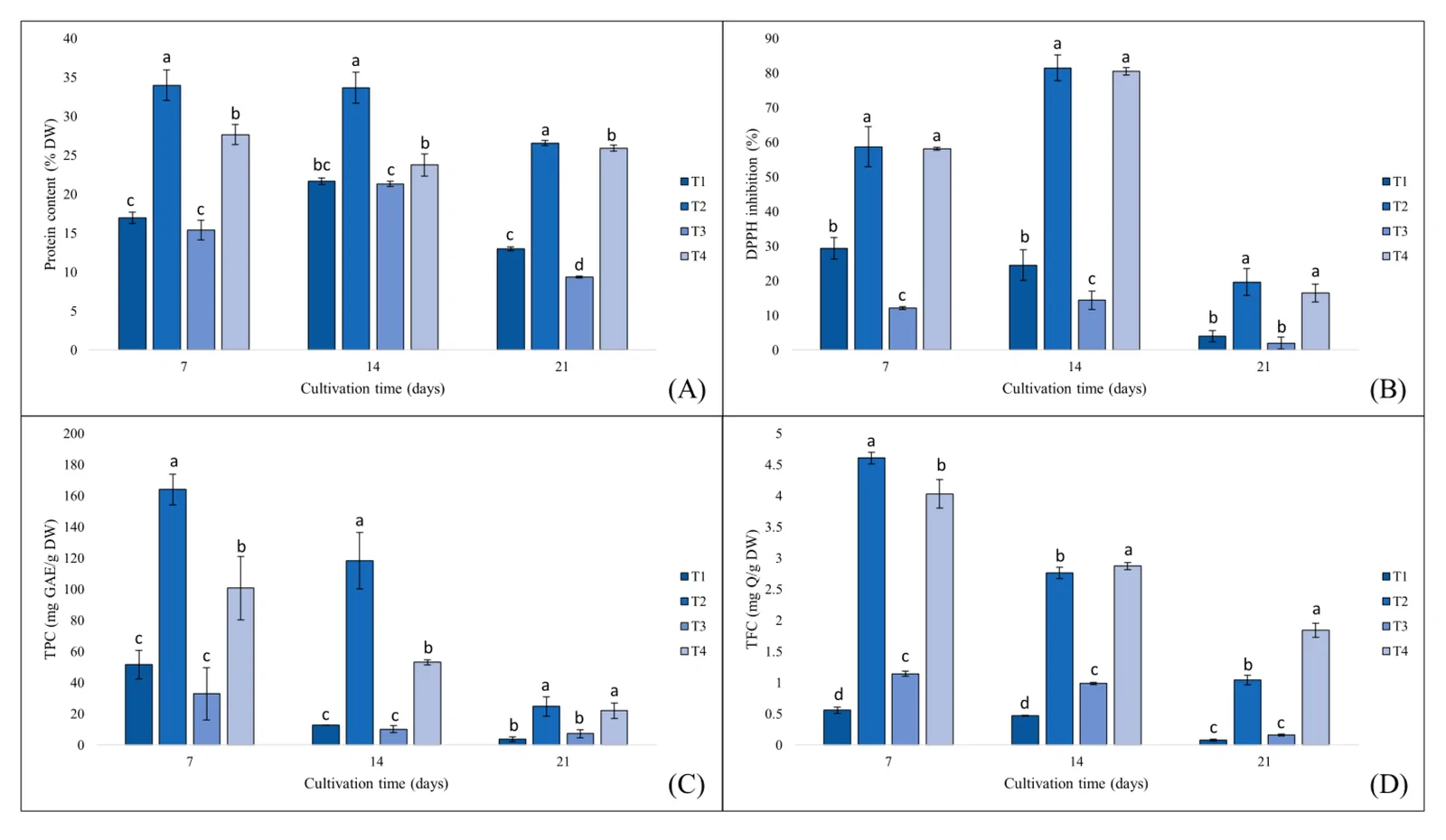
Biochemical characteristics of *Wolffia* spp. under different treatments during the cultivation period: (**A**) protein content (% DW), (**B**) DPPH radical activity (% inhibition), (**C**) total phenolic content (TPC; mg GAE g^−1^ DW), and (**D**) total flavonoid content (TFC; mg QE g^−1^ DW) measured at 7, 14, and 21 DAT. T1 (control), T2 (100% chemical fertilizer), T3 (crude extract containing 0.5 ppm IAA), and T4 (crude extract containing 0.5 ppm IAA combined with 50% chemical fertilizer). Bars represent the mean ± standard deviation (n = 3). Different letters above the bars indicate significant differences among treatments within the same sampling time according to the LSD test (*p* ≤ 0.05). (Detailed results are shown in Supplementary Table S2).

Protein content in watermeal differed significantly among treatments at all the sampling times (*p* ≤ 0.01). Overall, T2 consistently showed the highest protein content across all time points, followed by T4, while T1 and T3 remained lower. The highest protein content peaked at 14 DAT and declined thereafter in all the treatments. At 21 DAT, T2 and T4 showed comparable protein levels, both higher than T1 and T3 (Figure 6A).

#### 2.5.3. Total Phenolic Content (TPC)

Total phenolic content differed significantly among treatments at all the sampling times (*p* ≤ 0.01). T2 consistently exhibited the highest TPC, reaching 164.13 ± 9.82 mg GAE g^−1^ at 7 days and remaining the highest at 14 days. T4 showed the second-highest values, while T1 and T3 were consistently lower and not significantly different. TPC decreased markedly in all the treatments by 21 days, with T2 and T4 showing comparable levels (Figure 6C).

#### 2.5.4. Total Flavonoid Content (TFC)

Total flavonoid content differed significantly among treatments (*p* ≤ 0.01). At 7 days, T2 showed the highest TFC (4.61 ± 0.10 mg QE g^−1^), followed by T4. At 14 days, T4 slightly exceeded T2, and both treatments remained significantly higher than T1 and T3. By 21 days, TFC declined in all the treatments; however, T4 maintained the highest value (1.84 ± 0.12 mg QE g^−1^), followed by T2, while T1 remained the lowest throughout (Figure 6D).

#### 2.5.5. Antioxidant Activity (DPPH Radical Scavenging Activity)

DPPH radical scavenging activity differed significantly among treatments (*p* ≤ 0.01). T2 and T4 consistently showed the highest antioxidant activity, with values around 58.73 ± 5.78% and 58.18 ± 1.30% at 7 days and peaking at 81.52 ± 3.76% in T2 at 14 days. The activity declined in all the treatments by 21 days, although T2 and T4 remained higher than T1 and T3 (Figure 6B).

## 3. Discussion

This study successfully isolated bacterial strains from watermeal and screened them for their ability to produce IAA, one of the most important phytohormones involved in plant growth regulation. Among the isolates obtained, isolate W1/6 showed the highest IAA production. Based on 16S rRNA gene sequence analysis, isolate W1/6 was identified as *K. variicola*. Members of the genus *Klebsiella* have been widely used for plant growth promotion, particularly through IAA production [5]. Response surface methodology successfully optimized IAA production by *K. variicola* W1/6, and the developed model showed good predictive capability as indicated by the high coefficient of determination (R^2^ = 0.9213). These results suggest that the selected culture conditions were suitable for maximizing bacterial IAA production, thereby supporting its subsequent application in watermeal cultivation.

The combination of 50% chemical fertilizer and crude extract containing 0.5 ppm IAA resulted in rapid bud emergence and active vegetative propagation. At 14 DAT, this combined treatment produced biomass comparable to that of the full fertilizer treatment. Notably, at 21 DAT under greenhouse conditions, the combined treatment produced significantly higher fresh biomass than the full fertilizer treatment (78.61 ± 4.75 vs. 58.26 ± 12.42 g tray^−1^), while dry biomass remained statistically similar between the two treatments. This finding suggests that the bacterial-derived IAA extract may have prolonged vegetative growth and frond proliferation when combined with a reduced fertilizer supply, allowing continued fresh biomass accumulation during the later cultivation stage. In contrast, the application of crude IAA alone did not significantly increase biomass compared with the full chemical fertilizer treatment. This finding is consistent with previous findings that auxin mainly functions as a growth regulator rather than a substitute for nutrients [3,6]. Although auxin stimulates cell division and expansion, its growth-promoting effects depend on adequate nutrient availability to sustain plant metabolism and biomass accumulation. Similar positive effects of auxin and mineral nutrition have been reported in various crop species, where exogenous IAA promoted plant growth and biomass accumulation under reduced fertilizer application [7,8]. Auxin-mediated stimulation of frond development may increase the photosynthetically active surface and support plant growth under reduced fertilizer availability, although nutrient uptake was not directly evaluated in the present study [2,3].

Interestingly, chlorophyll levels in the combined treatment were nearly comparable to those in the full fertilizer treatment by Day 14 and Day 21, suggesting that the IAA-containing crude extract derived from *K. variicola* W1/6 helped maintain chlorophyll content under reduced fertilizer application, which may have supported photosynthetic performance. Previous studies have shown that auxin can promote chloroplast development and delay chlorophyll degradation, which may explain the sustained chlorophyll content observed in the present study [2,3]. The decline in chlorophyll content observed at Day 21 across treatments may be associated with developmental changes and shifts in metabolic allocation during rapid growth [9]. Similarly, the combined treatment maintained protein content at levels comparable to those of the full fertilizer treatment despite a 50% reduction in fertilizer input. Because proteins are essential components of photosynthetic enzymes, metabolic pathways, and cellular structures, maintaining protein accumulation is likely to support continued carbon assimilation, cellular metabolism, and plant growth. This observation is consistent with previous studies reporting that IAA-producing PGPR promoted protein accumulation in plants [7,10].

Secondary metabolite levels, including total phenolic compounds (TPC), flavonoid content (TFC), and antioxidants, also changed dynamically during growth. Both TPC and TFC were observed during the early stages, particularly in nutrient-supplemented treatments. At 21 DAT, the combined treatment maintained TPC, TFC, and antioxidant activity at levels comparable to those of the full fertilizer treatment despite a 50% reduction in fertilizer input. This finding suggests that while mineral nutrients were likely essential for supporting early plant establishment, the crude extract containing IAA may have helped sustain the accumulation of secondary metabolites during later developmental stages. The maintenance of biomass, chlorophyll, and protein accumulation, together with the decline in phenolic content at the later growth stage, suggests a developmental shift in carbon allocation from secondary metabolism toward primary metabolic processes required for rapid growth [11].

Overall, the findings demonstrate that crude IAA extract derived from *K. variicola* can effectively complement reduced chemical fertilizer input in watermeal cultivation. Although the crude extract alone did not achieve growth comparable to that of the full fertilizer treatment, its combined application with 50% chemical fertilizer maintained biomass production, chlorophyll and protein accumulation, as well as secondary metabolite content at levels comparable to those obtained with the recommended fertilizer rate. These results indicate that bacterial-derived IAA can function as an effective biostimulant when used in combination with mineral nutrients, rather than as a substitute for fertilizer. Consequently, integrating microbial-derived IAA with reduced fertilizer application represents a promising strategy for decreasing chemical fertilizer inputs while maintaining plant growth and physiological quality. This approach may contribute to more sustainable watermeal production by reducing reliance on chemical fertilizers without compromising crop performance.

The comparable growth observed in watermeal treated with crude IAA extract combined with 50% of the recommended fertilizer rate suggests the potential to reduce fertilizer inputs while maintaining biomass production. These findings indicate that bacterial-derived IAA may have potential as a biostimulant in watermeal cultivation systems. However, before large-scale implementation, further studies are required to optimize fermentation and extraction processes, ensure product stability and consistent IAA concentrations, evaluate economic feasibility, and validate the growth-promoting effects under commercial cultivation conditions and multiple production cycles.

## 4. Materials and Methods

### 4.1. Sources of Plant Material

The watermeal (Pham) samples used in this study were obtained from farms in Kalasin, Roi Et, and Bangkok provinces, Thailand.

### 4.2. Isolation of Bacteria

Bacteria were isolated from fresh watermeal by separating them into two groups: epiphytic bacteria residing on the cell surface and endophytic bacteria located inside the plant tissues. To isolate epiphytic bacteria, watermeal samples were gently rinsed with sterile distilled water. Approximately 1 g (wet weight) of each sample was transferred into 20 mL of nutrient broth (NB) (HiMedia Laboratories Pvt. Ltd., Mumbai, India) and incubated at 30 °C for 24 h with shaking at 150 rpm. Endophytic bacteria were isolated using a modified surface sterilization method described by Agarwal and Sheikh (2025) [12]. Briefly, the samples were immersed in 70% ethanol for 3 min, followed by treatment with 3% sodium hypochlorite for 2 min, and then immersed again in 70% ethanol for 30 s. The samples were subsequently rinsed three times with sterile distilled water. To confirm the effectiveness of sterilization, the final rinse water was plated onto nutrient agar (NA). Mechanical lysis was then performed to release the endophytic bacteria. The resulting homogenates were serially diluted (10-fold) using sterile distilled water. An aliquot of 100 µL from each dilution was spread onto NA plates. The plates were incubated at 30 °C for 24 h to allow bacterial growth. Individual colonies were selected based on distinct morphological, size, and color characteristics and streaked onto fresh NA plates to obtain pure isolates. The purified isolates were preserved in glycerol stocks and stored at −40 °C for further experimentation.

### 4.3. Screening of Bacteria for Indole-3-Acetic Acid (IAA) Production

All the bacterial isolates were determined to produce IAA using a colorimetric assay based on Salkowski’s method [13]. Initially, bacterial isolates were grown on NA at 30 °C for 24 h. A single colony from each isolate was then inoculated into 20 mL of NB to prepare the starter culture. Subsequently, 2 mL of EPB inoculum was added to NB supplemented with and without 0.2% (*w*/*v*) L-tryptophan. The cultures were incubated at 30 °C for 24 h with shaking at 150 rpm. After incubation, the cultures were centrifuged at 4000 rpm for 10 min to obtain the cell-free supernatant. One milliliter of the supernatant was mixed with 2 mL of Salkowski reagent, which consisted of 2 mL of 0.5 M FeCl_3_ and 98 mL of ACS-grade perchloric acid. The reaction mixtures were incubated at room temperature in the dark for 30 min. The development of a pink color indicated the presence of indole-related compound (IRC) production. Absorbance was measured at 530 nm using a spectrophotometer (Hitachi U-5100, Hitachi High-Technologies Corp., Tokyo, Japan). A standard calibration curve was prepared using pure IAA (Sigma-Aldrich, St. Louis, MI, USA), and the IAA concentration (µg/mL) in each sample was calculated based on the standard curve.

### 4.4. Identification of Bacteria

The most effective strain for producing IAA was selected for identification based on 16S rDNA sequences. Genomic DNA was extracted using the freeze–thaw method in lysis buffer, as modified by [14], and the lysates were used directly as templates for PCR amplification. Universal primers 27F (AGAGTTTGATCMTGGCTCAG) and 1492R (TACGGYTACCTTGTTACGACTT) were used for amplification [15]. PCR was carried out with an initial denaturation at 95 °C for 4 min, followed by 30 cycles of denaturation at 95 °C for 30 s, annealing at 55 °C for 45 s, and extension at 72 °C for 1 min, with a final extension at 72 °C for 10 min. The PCR reaction mixture was confirmed by electrophoresis on a 1.5% agarose gel stained with RedSafe™ Nucleic Acid Staining Solution (iNtRON Biotechnology, Seoul, Korea). PCR products were purified using the MEGAquick-spin™ Plus Total Fragment DNA Purification Kit (iNtRON Biotechnology, Seoul, Korea). Purified PCR products were sequenced by U2Bio (Thailand) Co., Ltd., Bangkok, Thailand. The obtained nucleotide sequences were analyzed using the BLAST available on the National Center for Biotechnology Information (NCBI) website. The sequences of bacterial isolates were aligned together with closely related sequences retrieved from the GenBank database using the CLUSTAL W algorithm. Phylogenetic analysis was performed using MEGA version 11 software. The reliability of the phylogenetic tree was evaluated by bootstrap analysis with 1000 replications [16].

### 4.5. Determination of Optimal Conditions for IAA Production Using Response Surface Methodology (RSM)

Response surface methodology (RSM) was employed to optimize IAA production by the selected bacterial isolate. Based on preliminary single-factor experiments, three independent variables, initial pH, incubation time (days), and inoculum size (%*v*/*v*), were selected for optimization using a face-centered central composite design (CCD; α = 1) (Table 5). For all the RSM experiments, LB broth was supplemented with 0.2% (*w*/*v*) L-tryptophan. The experimental design, data analysis, and model construction were performed using Design-Expert software version 7.0. A total of 17 experimental runs were conducted, consisting of 8 factorial points, 6 axial points, and 3 center points (Table 6). All the experiments were performed independently to validate the effects of the selected variables on IAA production. Statistical analysis was carried out using analysis of variance (ANOVA), with statistical significance defined at *p* < 0.05.

#### 4.5.1. Extraction of Indole-Related Compounds

The extraction of indole-related compounds from bacterial culture supernatants was performed using a modified method from Patten and Glick [17]. The bacteria were cultured in 2000 mL Luria–Bertani (LB) Broth medium supplemented with 0.2% (*w*/*v*) L-tryptophan under optimized conditions (initial pH, incubation time, and inoculum size), as determined from the experimental results in Section 4.5, to identify the optimal conditions for IAA production. The cultures were grown on a rotary shaker at 250 rpm and 30 °C. After incubation, the supernatant was collected by centrifugation at 10,000× *g* for 5 min. Final bacterial biomass was not determined before extraction. The supernatant containing IAA was acidified to pH 4.0 with 1 N hydrochloric acid (HCl) and extracted twice with an equal volume of ethyl acetate. The combined ethyl acetate fractions were evaporated to dryness using a rotary evaporator (Buchi R-210, Flawil, Switzerland). The resulting crude extract was dissolved in methanol and stored at −20 °C until further analysis.

#### 4.5.2. Identification of a Bacterial IAA Biosynthesis Pathway

Quantification and identification of the extraction of indole-related compounds from bacteria were performed using high-performance liquid chromatography (HPLC) based on a modified method of Szkop & Bielawski [18]. The dried crude extracts were dissolved in methanol and filtered through a 0.22 µm pore-size membrane filter. Chromatographic separation was achieved using gradient elution. Solvent A consisted of 2.5% (*v*/*v*) acetic acid and 97.5% (*v*/*v*) deionized water, with the pH adjusted to 3.8 using 10 mol/L KOH. Solvent B consisted of 80% (*v*/*v*) acetonitrile and 20% (*v*/*v*) deionized water. The mobile phase composition was initially set at 80:20 (A:B), changed to 50:50, then to 0:100, and finally returned to 80:20 at 25, 31, and 33 min, respectively. All the mobile phases were filtered through a 0.22 µm nylon membrane filter before use. For HPLC analysis, a 20 µL aliquot of each sample was injected into an HPLC system equipped with a reverse-phase C18 Inertsil ODS-3 column (4.6 × 250 mm) (Varian, Torrance, CA). maintained at 40 °C and a Shimadzu SPD-M20A diode array detector (Shimadzu Corporation, Kyoto, Japan). The total run time was 40 min, with a mobile phase flow rate of 0.5 mL/min. Detection was performed at wavelengths of 280 and 350 nm. Identification of indole-related compounds was achieved by comparing retention times and UV spectra of sample peaks with those of authentic standards, including L-tryptophan (L-Trp), indole-3-acetic acid (IAA), indole-3-acetamide (IAM), tryptamine (TAM), indole-3-acetonitrile (IAN), and indole-3-lactic acid (ILA). Compound identities were further confirmed by co-injection with the corresponding standards.

### 4.6. The Effect of Combining Chemical Fertilizer with IAA-Containing Crude Extract on the Growth Promotion of Watermeal Under Laboratory and Greenhouse Conditions

The effect of crude extract containing IAA on promoting the growth of watermeal was evaluated under laboratory conditions using a plate assay. The experiment consisted of five treatments, including (Control) sterile distilled water, (100%) full dose of chemical fertilizer (NPK of 15-15-15) (Chia Tai Co., Ltd., Bangkok, Thailand), (CE + 100%) crude extract containing 0.5 ppm IAA and full dose of chemical fertilizer, (CE + 50%) crude extract containing 0.5 ppm IAA and half dose (50%) of chemical fertilizer, (CE + 25%) crude extract containing 0.5 ppm IAA and quarter dose (25%) of chemical fertilizer. The IAA concentration in the crude extract was determined using the Salkowski colorimetric assay. Each plate was filled with 20 mL of the respective treatment solution. Watermeal fronds were surface sterilized by immersion in 70% ethanol for 3 min, followed by 3% sodium hypochlorite for 2 min, then 70% ethanol for 30 s, and a final rinsing with sterilized distilled water three times. Ten fronds were aseptically transferred to each plate and maintained under modified growth conditions [19]. The cultures were incubated at 25 ± 2 °C under full-spectrum LED lighting (pink LED lamps providing a combination of red and blue light) at a light intensity of 8000–10,000 lux, using a photoperiod of 16 h light and 8 h dark for 10 days. To investigate the morphological changes in watermeal fronds, a laboratory experiment was conducted under the same culture conditions described above. Four treatments were evaluated: (T1) sterile distilled water (control), (T2) a full dose (100%) of chemical fertilizer (NPK 15-15-15), (T3) crude extract containing 0.5 ppm IAA, and (T4) crude extract containing 0.5 ppm IAA combined with a half dose (50%) of chemical fertilizer. One surface-sterilized frond was aseptically transferred to each Petri dish and maintained under the same culture conditions. Morphological changes were observed under a stereomicroscope throughout the cultivation period (0–7 days).

In greenhouse conditions, the experiment was carried out at the Agronomy Farm, Khon Kaen University, Thailand. A factorial randomized complete block design (RCBD) was employed in the experiment with four treatments, including (1) tap water (control), (2) full dose (100%) of chemical fertilizer (NPK 15–15–15), (3) crude extract containing 0.5 ppm IAA, and (4) crude extract containing 0.5 ppm IAA combined with half dose (50%) of chemical fertilizer. Each treatment consisted of nine independent replicates. All the treatments were cultured in plastic trays (27 × 42.5 × 9.5 cm) containing 10 g of fresh watermeal and 5 L of water. The crude extract containing 0.5 ppm IAA and chemical fertilizer was applied once at the beginning of the experiment and was not reapplied during the 21-day experimental period. Watermeal samples were collected at 7, 14, and 21 DAT to evaluate growth parameters. Because destructive sampling was employed, different trays were harvested at each sampling time. Therefore, three biological replicates (n = 3) were obtained for each treatment at each sampling time, and each tray was considered an experimental unit for statistical analysis.

#### 4.6.1. Determination of Chlorophyll Content and Biomass Parameters

Watermeal growth parameters were measured at 7, 14, and 21 days after cultivation. Biomass (fresh and dry weight of watermeal) was recorded. Dry weight was determined after oven-drying at 50 °C. The chlorophyll content was determined following the method of Arnon [20] with some modifications. Fresh watermeal was washed, ground, and 1 g of the sample was extracted with 80% acetone. The extract was centrifuged at 4000 rpm for 10 min, and the supernatant was collected. Absorbance values were measured at 663 and 645 nm using a spectrophotometer (Hitachi High-Tech Science Corporation, Tokyo, Japan). Chlorophyll contents were calculated using the following equations:

Chlorophyll a content = (12.7 × Abs663) − (2.69 × Abs645)

Chlorophyll b content = (22.9 × Abs645) − (4.68 × Abs663)

Total chlorophyll content = (20.2 × Abs645) + (8.02 × Abs663)

#### 4.6.2. Determination of Protein Concentration

##### Alkaline Extraction (ALK)

The dry weight of watermeal was ground and sieved through a 40-mesh sieve. Protein extraction was carried out using alkaline extraction followed by isoelectric precipitation with slight modifications to the method described by Siriwat et al. [21]. Briefly, the powder was mixed with distilled water (1:30 *w*/*v*), and the pH was adjusted to 10. The mixture was stirred for 30 min and then centrifuged at 10,000 rpm at 4 °C for 15 min. The supernatant was collected and adjusted to pH 3 to precipitate proteins. The precipitate was recovered by centrifugation and then adjusted to pH 7 before freeze-drying.

##### Lowry Method

Protein content was determined using the method of Lowry et al. [22]. Standard solutions were prepared using bovine serum albumin (BSA). For analysis, 40 µL of the sample was mixed with 200 µL of the Lowry working reagent and incubated for 10 min. Subsequently, 20 µL of 1 M Folin–Ciocalteu reagent was added and incubated for 30 min at room temperature. Absorbance was measured at 760 nm.

#### 4.6.3. Determination of Total Phenolic Content (TPC)

Total phenolic content (TPC) was determined using the Folin–Ciocalteu colorimetric method, modified from Siriamornpun et al. [23]. An aliquot (125 µL) of the extract was mixed with 250 µL of Folin–Ciocalteu reagent and 3 mL of distilled water, and the mixture was allowed to stand for 6 min. Subsequently, 2.5 mL of 7% sodium carbonate (Na_2_CO_3_) was added, and the mixture was incubated at room temperature for 90 min. The absorbance was measured at 760 nm using a spectrophotometer. The results were expressed as milligrams of gallic acid equivalents per gram of dry weight (mg GAE/g DW).

#### 4.6.4. Determination of Total Flavonoid Content (TFC)

Total flavonoid content (TFC) was determined using the aluminum chloride colorimetric method of Prommuak et al. [24], with quercetin as the standard. Quercetin standard solutions were prepared at concentrations of 25–400 mg/mL in 80% ethanol (*v*/*v*). For the analysis, 0.5 mL of extract or standard solution was mixed with 1.5 mL of 95% ethanol, followed by the addition of 0.1 mL of 10% aluminum chloride (AlCl_3_), 0.1 mL of 1 M potassium acetate, and 2.8 mL of distilled water. The mixture was incubated at room temperature for 30 min. The absorbance was measured at 415 nm using a spectrophotometer. A blank was prepared by replacing the aluminum chloride solution with distilled water. The results were expressed as milligrams of quercetin equivalents per gram of dry weight (mg QE/g DW).

#### 4.6.5. Determination of Antioxidant Activity

Antioxidant activity was determined using the DPPH radical scavenging assay according to the method, modified from Brand-Williams [25]. Briefly, a DPPH solution (0.05 mM) was prepared in methanol and stored at −20 °C until use. The extract (0.5 mL) was mixed with 4.5 mL of DPPH solution and incubated in the dark at room temperature for 30 min. Absorbance was measured at 517 nm using a spectrophotometer (Hitachi U-5100, Japan). A calibration curve was constructed using Trolox. DPPH scavenging activity was calculated as

$$\%\;Inhibition=\left[\frac{Absorbance\;of\;DPPH-Absorbance\;of\;sample}{Absorbance\;of\;DPPH-Absorbance\;of\;blank}\right]\times 100$$

### 4.7. Statistical Analysis

All the experiments were performed in three replicates. Data were analyzed using Statistix version 10.0. Analysis of variance (ANOVA) was performed, and mean comparisons were conducted using the least significant difference (LSD) test at *p* ≤ 0.05.

## 5. Conclusions

This study demonstrated that an endophytic bacterium, *K. variicola* W1/6, isolated from watermeal, is a promising producer of indole-3-acetic acid (IAA). IAA production was successfully optimized using response surface methodology, and HPLC analysis suggested that IAA biosynthesis predominantly proceeded via the indole-3-acetamide (IAM) pathway. Notably, application of crude extract containing 0.5 ppm IAA combined with 50% chemical fertilizer achieved watermeal growth, protein accumulation, and secondary metabolite content at levels comparable to those with the full fertilizer treatment. These findings support the use of microbial-derived IAA as a promising biostimulant for reducing chemical fertilizer inputs and promoting more sustainable watermeal cultivation. Further studies are needed to evaluate its performance under larger-scale cultivation systems and to investigate nutrient dynamics during cultivation while assessing its agronomic performance and economic feasibility.

## Figures and Tables

**Table 1:** Analysis of variance (ANOVA) for indole-3-acetic acid (IAA) production by *K. variicola* W1/6.

Source	Sum of Squares	df	Mean Square	F-Value	*p*-Value
Model	13,078.2	9	1453.13	9.11	0.0041
A-pH	1585.33	1	1585.33	9.94	0.0161
B-Incubation time	2963.91	1	2963.91	18.58	0.0035
C-Inoculum size	1543.56	1	1543.56	9.68	0.0171
AB	2385.68	1	2385.68	14.96	0.0062
AC	609.18	1	609.18	3.82	0.0916
BC	1510.03	1	1510.03	9.47	0.0179
A^2^	66.16	1	66.16	0.4148	0.54
B^2^	232.44	1	232.44	1.46	0.2665
C^2^	599.17	1	599.17	3.76	0.0938
Residual	1116.42	7	159.49		
Lack of Fit	650.07	5	130.01	0.5576	0.7413
Pure Error	466.35	2	233.18		
Cor Total	14,194.6	16			

**Table 2:** Experimental design and results of the face-centered central composite design (CCD; α = 1).

Experiment	Code Levels			IAA Production (µg/mL)	
	A	B	C	Actual Value	Predicted Value
1	7.00 (0)	1.00 (−1)	10.00 (0)	127.64	130.02
2	7.00 (0)	3.00 (0)	15.00 (1)	155.48	154.02
3	5.00 (−1)	1.00 (−1)	15.00 (1)	120.28	115.13
4	9.00 (1)	3.00 (0)	10.00 (0)	154.72	164.18
5	7.00 (0)	3.00 (0)	5.00 (−1)	126.95	129.18
6	9.00 (1)	1.00 (−1)	15.00 (1)	152.52	157.40
7	5.00 (−1)	5.00 (1)	5.00 (−1)	146.87	141.80
8	7.00 (0)	5.00 (1)	10.00 (0)	166.07	164.46
9	9.00 (1)	1.00 (−1)	5.00 (−1)	133.13	122.52
10	9.00 (1)	5.00 (1)	15.00 (1)	138.5	129.81
11	7.00 (0)	3.00 (0)	10.00 (0)	164.37	156.56
12	7.00 (0)	3.00 (0)	10.00 (0)	139.52	156.56
13	5.00 (−1)	1.00 (−1)	5.00 (−1)	36.86	45.35
14	7.00 (0)	3.00 (0)	10.00 (0)	167.32	156.56
15	5.00 (−1)	5.00 (1)	15.00 (1)	146.21	156.62
16	9.00 (1)	5.00 (1)	15.00 (1)	144.94	149.90
17	5.00 (−1)	3.00 (0)	10.00 (0)	147.68	138.99

**Table 3:** Number of fronds and frond size of watermeal grown on plates under laboratory conditions at 10 days after transplantation.

Treatment	Number of Watermeal	Size of Watermeal (mm)
Control	47.33 ± 6.81 b	0.58 ± 0.06
100%	74.00 ± 2.65 a	0.58 ± 0.09
CE + 100%	74.00 ± 5.66 a	0.63 ± 0.08
CE + 50%	73.00 ± 4.24 a	0.60 ± 0.05
CE + 25%	53.00 ± 3.61 b	0.55 ± 0.05
%CV	12.97	11.84
F-test	*	ns

Means followed by at least one common letter were not significantly different according to LSD test, ns: not significantly, *: Significant difference at *p* ≤ 0.05; (100%: full dose (100%) of chemical fertilizer (NPK of 15-15-15); CE + 100, 50 and 25%: crude extract containing 0.5 ppm IAA and full dose (100%), half dose (50%) and quarter dose (25%) of chemical fertilizer).

**Table 4:** The results demonstrated the chlorophyll content and biomass of watermeal throughout the cultivation period on days 7, 14 and 21 under greenhouse conditions.

Days	Treatment	Chl a (mg g^−1^ FW)	Chl b (mg g^−1^ FW)	Total Chl (mg g^−1^ FW)	FW (g tray^−1^)	DW (g tray^−1^)
7	T1	12.55 ± 0.10 c	7.95 ± 0.52 b	20.49 ± 0.57 b	18.36 ± 0.32 b	0.63 ± 0.03 a
	T2	21.36 ± 3.17 a	13.12 ± 2.89 a	32.63 ± 7.02 a	33.87 ± 5.01 a	0.45 ± 0.01 b
	T3	12.25 ± 0.49 c	7.16 ± 1.33 b	18.51 ± 1.83 b	19.29 ± 0.38 b	0.68 ± 0.07 a
	T4	17.41 ± 0.51 b	9.41 ± 2.12 b	25.65 ± 3.42 b	35.31 ± 1.45 a	0.75 ± 0.10 a
	%CV	10.23	20.52	16.53	9.80	10.12
	F-test	**	*	*	**	**
14	T1	8.27 ± 0.17 c	3.71 ± 0.11 b	12.46 ± 1.00 c	27.61 ± 2.05 b	1.45 ± 0.08 b
	T2	13.78 ± 1.67 a	7.24 ± 0.53 a	21.92 ± 1.14 a	68.56 ± 5.38 a	2.17 ± 0.31 a
	T3	10.24 ± 0.01 b	7.28 ± 0.34 a	17.51 ± 0.35 b	26.09 ± 1.33 b	1.34 ± 0.11 b
	T4	10.24 ± 0.80 b	6.17 ± 1.79 a	16.17 ± 2.54 b	65.24 ± 5.07 a	2.48 ± 0.14 a
	%CV	8.75	15.57	8.76	8.31	9.79
	F-test	**	**	**	**	**
21	T1	4.53 ± 0.28 a	2.33 ± 0.40 b	6.86 ± 0.64 b	24.95 ± 4.91 c	1.23 ± 0.18 b
	T2	3.6 ± 0.13 b	4.54 ± 1.42 a	10.05 ± 1.07 a	58.26 ± 12.42 b	2.09 ± 0.28 a
	T3	4.66 ± 0.22 a	2.85 ± 0.38 b	6.67 ± 0.32 b	26.26 ± 7.40 c	1.29 ± 0.33 b
	T4	4.14 ± 0.56 ab	3.45 ± 0.57 b	7.85 ± 0.89 b	78.61 ± 4.75 a	2.53 ± 0.00 a
	%CV	7.98	24.75	9.97	17.00	13.09
	F-test	**	*	**	**	**

Values are presented as mean ± SD (n = 3). Different letters indicate significant differences among values within the same column by the LSD test. Values are means of triplicate. * Significant difference at *p* < 0.05; ** Significant difference at *p* < 0.01. Chl: chlorophyll content; FW: fresh weight; and DW: dry weight.

**Table 5:** Selected factors and their levels for the face-centered central composite design (CCD; α = 1).

Independent Variables	Symbols	Code Levels		
		−1	0	+1
Initial pH	A	5.00	7.00	9.00
Incubation time (days)	B	1.00	3.00	5.00
Inoculum size (%*v*/*v*)	C	5.00	10.00	15.00

**Table 6:** Experimental design and results of the face-centered central composite design (CCD; α = 1).

Experiment	A	B	C
1	7.00 (0)	1.00 (−1)	10.00 (0)
2	7.00 (0)	3.00 (0)	15.00 (1)
3	5.00 (−1)	1.00 (−1)	15.00 (1)
4	9.00 (1)	3.00 (0)	10.00 (0)
5	7.00 (0)	3.00 (0)	5.00 (−1)
6	9.00 (1)	1.00 (−1)	15.00 (1)
7	5.00 (−1)	5.00 (1)	5.00 (−1)
8	7.00 (0)	5.00 (1)	10.00 (0)
9	9.00 (1)	1.00 (−1)	5.00 (−1)
10	9.00 (1)	5.00 (1)	15.00 (1)
11	7.00 (0)	3.00 (0)	10.00 (0)
12	7.00 (0)	3.00 (0)	10.00 (0)
13	5.00 (−1)	1.00 (−1)	5.00 (−1)
14	7.00 (0)	3.00 (0)	10.00 (0)
15	5.00 (−1)	5.00 (1)	15.00 (1)
16	9.00 (1)	5.00 (1)	15.00 (1)
17	5.00 (−1)	3.00 (0)	10.00 (0)

## Data Availability

The datasets obtained and analyzed in the current study are available from the corresponding author upon reasonable request.

## Supplementary Materials

The following supporting information can be downloaded at https://www.mdpi.com/article/10.3390/plants15172642/s1. Table S1: IAA production of bacterial isolates from *Wolffia* spp.; Table S2: Biochemical characteristics of *Wolffia* sp. under different treatments during the cultivation period: effects of different treatments on protein content, total phenolic content (TPC), total flavonoid content (TFC), and DPPH radical-scavenging activity of watermeal at 7, 14, and 21 days after treatment under greenhouse conditions.

## References

[1] Appenroth K.-J., Sree K.S., Böhm V., Hammann S., Vetter W., Leiterer M., Jahreis G. (2017). Nutritional Value of Duckweeds (Lemnaceae) as Human Food. Food Chem..

[2] Seephua N., Boonarsa P., Li H., Thammapat P., Siriamornpun S. (2025). Nutritional Composition and Bioactive Profiles of Farmed and Wild Watermeal (*Wolffia globosa*). Foods.

[3] Taiz L., Zeiger E., Moller I.M., Murphy A. (2015). Plant Physiology and Development.

[4] Glick B.R. (2012). Plant Growth-Promoting Bacteria: Mechanisms and Applications. Scientifica.

[5] Kim A.-Y., Shahzad R., Kang S.-M., Seo C.-W., Park Y.-G., Park H.-J., Lee I.-J. (2017). IAA-Producing Klebsiella Variicola AY13 Reprograms Soybean Growth during Flooding Stress. J. Crop Sci. Biotechnol..

[6] Davies P.J. (2010). Plant Hormones.

[7] Spaepen S., Vanderleyden J., Remans R. (2007). Indole-3-Acetic Acid in Microbial and Microorganism-Plant Signaling. FEMS Microbiol. Rev..

[8] Vessey J.K. (2003). Plant Growth Promoting Rhizobacteria as Biofertilizers. Plant Soil.

[9] Lambers H., Chapin F.S., Pons T.L. (2008). Plant Physiological Ecology.

[10] Bhattacharyya P.N., Jha D.K. (2012). Plant Growth-Promoting Rhizobacteria (PGPR): Emergence in Agriculture. World J. Microbiol. Biotechnol..

[11] Herms D.A., Mattson W.J. (1992). The Dilemma of Plants: To Grow or Defend. Q. Rev. Biol..

[12] Agarwal M., Sheikh M.B. (2025). Isolation and Functional Characterization of Endophytic Bacteria from Muscadine Grape Berries: A Microbial Treasure Trove. Cells.

[13] Khamsuk K., Dell B., Pathom-aree W., Pathaichindachote W., Suphrom N., Nakaew N., Jumpathong J. (2024). Screening Plant Growth-Promoting Bacteria with Antimicrobial Properties for Upland Rice. J. Microbiol. Biotechnol..

[14] Queipo-Ortuño M.I., De Dios Colmenero J., Macias M., Bravo M.J., Morata P. (2008). Preparation of Bacterial DNA Template by Boiling and Effect of Immunoglobulin G as an Inhibitor in Real-Time PCR for Serum Samples from Patients with Brucellosis. Clin. Vaccine Immunol..

[15] He X., Yuan H., Li Y., Yang C. (2026). Endophytic Plant Growth Promoting Bacteria from Two Halophytes Improve Wheat Performance under Salt Stress. Front. Plant Sci..

[16] Sohpal V.K., Dey A., Singh A. (2010). MEGA Biocentric Software for Sequence and Phylogenetic Analysis: A Review. Int. J. Bioinform. Res. Appl..

[17] Gang S., Sharma S., Saraf M., Buck M., Schumacher J. (2019). Analysis of Indole-3-Acetic Acid (IAA) Production in Klebsiella by LC-MS/MS and the Salkowski Method. Bio-Protocol.

[18] Szkop M., Bielawski W. (2013). A Simple Method for Simultaneous RP-HPLC Determination of Indolic Compounds Related to Bacterial Biosynthesis of Indole-3-Acetic Acid. Antonie Leeuwenhoek.

[19] Prosridee K., Oonsivilai R., Tira-aumphon A., Singthong J., Oonmetta-aree J., Oonsivilai A. (2023). Optimum Aquaculture and Drying Conditions for *Wolffia arrhiza* (L.) Wimn. Heliyon.

[20] Arnon D.I. (1949). Copper enzymes in isolated chloroplasts. Polyphenoloxidase in *Beta vulgaris*. Plant Physiol..

[21] Siriwat W., Ungwiwatkul S., Unban K., Laokuldilok T., Klunklin W., Tangjaidee P., Potikanond S., Kaur L., Phongthai S. (2023). Extraction, Enzymatic Modification, and Anti-Cancer Potential of an Alternative Plant-Based Protein from *Wolffia globosa*. Foods.

[22] Lowry O., Rosebrough N., Farr A.L., Randall R. (1951). Protein measurement with the folin phenol reagent. J. Biol. Chem..

[23] Siriamornpun S., Kaewseejan N. (2017). Quality, Bioactive Compounds and Antioxidant Capacity of Selected Climacteric Fruits with Relation to Their Maturity. Sci. Hortic..

[24] Prommuak C., De-Eknamkul W., Shotipruk A. (2008). Extraction of Flavonoids and Carotenoids from Thai Silk Waste and Antioxidant Activity of Extracts. Sep. Purif. Technol..

[25] Leong L.P., Shui G. (2002). An Investigation of Antioxidant Capacity of Fruits in Singapore Markets. Food Chem..

