# Supplementary material for: Indole-3-Acetic Acid Production by Klebsiella variicola from Watermeal and Its Role in Enhancing Watermeal Growth
Source: Plants (Basel). 2026 Aug 28;15(17):2642. doi: 10.3390/plants15172642 (PMC13567710; doi:10.3390/plants15172642)
Supplement: Supplementary file 1 [file plants-15-02642-s001.zip › plants-4489338-supplementary.pdf]

Table S1: IAA production of bacterial isolates from *Wolffia* spp.

Location	Isolate no.	IAA production (μg/mL)	Location	Isolate no.	IAA production (μg/mL)
Kalasin (R1)	W1/1	3.621	Roi Et (R1)	W2/1	62.23
	W1/2	1.92		W2/2	1.19
	W1/3	1.09		W2/3	0.96
	W1/4	2.16		W2/4	1.30
	W1/5	2.16		WT2/1	0.80
	W1/6	106.34		WT2/2	1.43
	WT1/2	0.00		WT2/3	2.70
	WT1/3	87.47		WT2/4	0.72
	WT1/4	0.00		WT2/5	3.19
Kalasin (R2)	W4/2	0.00		WT2/6	0.96
	W4/3	20.93		WT2/7	4.72
	W4/4	58.82		WT2/8	8.24
	WT4/1	1.96	Bangkok (R1)	W3/1	20.44
	WT4/2	1.60		W3/2	1.55
	WT4/3	1.33		W3/3	1.94
	WT4/4	1.54		W3/4	0.83
				WT3/1	0.76
				WT3/2	1.39
				WT3/3	1.73

R1: first isolation and R2: second isolation

Table S2: Biochemical characteristics of *Wolffia sp*. under different treatments during the cultivation period: effects of different treatments on protein content, total phenolic content (TPC), total flavonoid content (TFC), and DPPH radical-scavenging activity of watermeal at 7, 14, and 21 days after treatment under greenhouse conditions.

Cultivation time (days)	Treatment	Protein content (% DW)	TPC (mg GAE g^{-1}DW)	TFC (mg QE g^{-1}DW)	DPPH inhibition (%)
7	T1	16.95±0.73c	51.63±9.25c	0.56±0.05d	29.36 ±3.08b
	T2	33.99±1.95a	164.13±9.82a	4.61±0.10a	58.73 ±5.78a
	T3	15.40±1.27c	32.88±16.82c	1.14±0.04c	12.12 ±0.41b
	T4	27.66±1.28b	100.79±20.33b	4.03±0.52b	58.18 ±1.30a
	%CV	5.86	16.96	10.35	8.45
	F-test	**	**	**	**
14	T1	21.67±0.41bc	12.88±0.00c	0.48±0.29d	24.50 ±4.41b
	T2	33.67±1.97a	118.29±18.07a	2.76±0.09b	81.52 ±3.76a
	T3	21.34±0.32c	10.17±2.19c	1.01±0.04c	14.37 ±2.69c
	T4	23.76±1.42b	53.08±1.57b	2.50±0.43a	80.49 ±1.09a
	%CV	4.95	18.8	15.64	6.45
	F-test	**	**	**	**
21	T1	12.97±0.25c	3.81±1.56b	0.08±0.02c	4.03 ±1.61b
	T2	26.57±0.34a	24.75±6.25a	1.04±0.08b	19.61 ±3.85a
	T3	9.38±0.13d	7.25±2.50b	0.16±0.01c	2.01 ±1.69b
	T4	25.91±0.39b	22.04±5.05a	1.84±0.12a	16.48 ±2.51a
	%CV	1.58	29.59	9.05	24.49
	F-test	**	**	**	**

Values are presented as mean ± SD (n = 3). Different lowercase letters within the same column and sampling time indicate significant differences among treatments according to the LSD test. * and ** indicate significant differences at $P < 0.05$ and $P < 0.01$, respectively. DW, dry weight; TPC, total phenolic content; TFC, total flavonoid content; DPPH, 2,2-diphenyl-1-picrylhydrazyl radical-scavenging activity.
